# Cross Sectional Characterization of Factors Associated with Pediatric HIV Status Disclosure in Southern Ethiopia

**DOI:** 10.1371/journal.pone.0132691

**Published:** 2015-07-13

**Authors:** Birkneh Tilahun Tadesse, Byron Alexander Foster, Yifru Berhan

**Affiliations:** 1 Department of Child Health, Hawassa University, College of Health Sciences, Hawassa, Ethiopia; 2 Department of Pediatrics, University of Texas Health Science Center at San Antonio, San Antonio, Texas, United States of America; Alberta Provincial Laboratory for Public Health/ University of Alberta, CANADA

## Abstract

**Background:**

Disclosure of HIV positive status to children and adolescents is a complex process. However, disclosure has been found to be associated with improved outcomes. The objective of the current study was to identify the predictors that facilitate disclosure of HIV status to children and adolescents and to study the reasons for non-disclosure.

**Methods:**

Interviews of caregivers and reviews of records were done to collect data on caregiver and child information and details regarding the disclosure status of children. Bivariate analysis was done to test the association between HIV status disclosure and different caregiver and child factors. To identify the independent predictors of disclosure, we did multivariable logistic regression.

**Results:**

A total of 177 children attending an HIV clinic were included. The mean age of the participants was 10.1 years (SD = 2.8), and about half (50.8%) were female. Most caregivers, 137 (77.8%) stated that disclosure of HIV status to children is important and should be done. However, disclosure had only been made to 59 (33.3%) of the participants. Child age more than 10 years [AOR = 6.7; 95%CI: 1.73–26.01], duration of HIV diagnosis of 5 years or more [AOR = 4.4; 95%CI: 1.26–15.06] and taking a zidovudin (AZT) based regimen [AOR = 3.5; 95%CI: 1.31–9.53] predicted HIV positive status disclosure. Additionally, length of treatment of caregivers of more than 14 years [AOR = 3.9; 95%CI: 1.07–14.61], disclosure of caregiver’s HIV status to children and/or others [AOR = 4.7; 95%CI: 1.19–18.74], and the child’s inquiry about their condition [AOR = 4.5; 95%CI: 1.16–17.43] increased the odds of disclosure.

**Conclusion:**

The rate of disclosure among HIV infected children in southern Ethiopia is low. Primarily time-based factors were associated with the probability of HIV positive status disclosure and a specific regimen which has not been found previously. Further qualitative research may elucidate more on these factors; educational strategies may address some of these determinants.

## Introduction

An estimated 3.2 million children were living with HIV at the end of 2013, mostly in sub-Saharan Africa. The majority of them acquire HIV from their mothers during pregnancy, birth or breastfeeding. With efficacious interventions, the risk of mother-to-child transmission can be reduced to 2%. However such interventions are still not widely accessible or available in most resource-limited countries where the burden of HIV is highest [1, 2]. HIV/AIDS affects the health and welfare of children and undermines hard-won gains in child survival in highly affected countries [3, 4].

With increased survival, one of the greatest psychosocial challenges that parents and caregivers of perinatally HIV-infected children face is disclosure of HIV serostatus to their infected children. HIV diagnosis disclosure entails communication about a potentially life threatening, stigmatized and transmissible illness and many caregivers fear that such communication may create distress for the child [5]. Disclosure of HIV diagnosis is not an isolated event but rather a step in the process of adjustment by a child, the caretakers and the community to an illness and the life challenges that is poses.

Factors associated with HIV serostatus non-disclosure have been explored, with corresponding explanatory theories proposed, since the early 1990s [6]. In the literature, the proportion of disclosed children ranges from 0 to 69.2%. Child’s age and perceived ability to understand the meaning of HIV infection, education level of the parent, openness about parental HIV status, and beliefs about children’s capacities have been found to be related to disclosure [7].

The socio-cultural profile in the developing world is different, and the rate of HIV status disclosure is not precisely known in this setting. To target educational interventions, a range of possible predictors to disclosure should be identified. Furthermore, while adherence to anti-retroviral therapy (ART) is widely recognized as an important goal, and the social context has a significant influence on adherence, there is little understanding of the impact of disclosure on ART adherence on the child’s functioning in society and school. As a first step towards an improved understanding of these relationships, the current study assessed the proportion and predictors of disclosure of HIV positive status to HIV infected children and adolescents attending the ART clinic of Hawassa University Referral Hospital in southern Ethiopia.

## Methods

### Ethical Consideration

Institutional Review Board (IRB) approval was obtained from Hawassa University College of Medicine and Health Sciences. Adults provided consent and children assent to participate in this study after a careful description of the objectives and purpose of the study. They were informed that they were free to not continue the interview/study at any time. To facilitate understanding, the questionnaires were interpreted into their local language or Amharic as appropriate. All questionnaires were kept anonymous.

### Design and Subjects

The study was conducted at Hawassa University Referral Hospital (HURH). The hospital is the largest in capacity in southern Ethiopia. Parents or caregivers of all children and adolescents infected with HIV attending the HIV/AIDS clinic were invited to participate in the study. A cross-sectional study was employed to measure the rate of HIV+ status disclosure, age at disclosure and to determine the factors associated with non-disclosure. All children between the ages of 5–18 years with their caregivers who consented to participate were included in the study. Children below the age of 5 years were not included as they are reasonably likely not to have been disclosed.

Data were collected by trained nurses working at the ART clinics with close supervision by the investigators. The healthcare professionals working in the pediatric HIV clinics were all trained on pediatric HIV care and treatment including potential disclosure. Detailed training on how to interview and complete the questionnaire including skip patterns was provided by the investigators. Children and adolescents were interviewed while they came for their regular visit at the clinic. Face to face interview was conducted between the caregiver and HIV+ status disclosed adolescents themselves.

For clinical and laboratory parameters, anthropometry and CD4 count values, we reviewed the records for each study participant. Structured, piloted questionnaires were used to collect data (S1 Questionnaire). The questionnaires included information regarding the child and the caregivers. The socio-demographic, HIV and ART details of pairs were obtained. For this study, disclosure was defined as an affirmative answer to the query: “Did you disclose your child’s HIV status to her/himself?”

### Statistical Analysis

Data were entered, cleaned and analyzed using SPSS for windows version 20 (IBM, USA) after checking for completeness. Descriptive statistics as numbers (percentage), mean (standard deviation) and median (range) were generated. Factors associated with non-disclosure were described using odds ratio (OR) and confidence intervals (CI) to test the presence and strength of association. Full data set available as a supplement (S1 Dataset).

## Results

There were 177 children and adolescents enrolled from the HIV clinic of Hawassa University Referral Hospital, and all were included in the analysis. The mean age of the participants was 10.1 years (SD = 2.8), 95%CI: 9.7–10.6. Nearly half (50.8%) of the participants were female. The majority (71.8%) of the participants were at elementary school or above. The majority were also first or second born in terms of birth order.

Diagnosis of HIV was made more than five years prior in 57.0% of participants. The WHO clinical stage of the participants at time of interview was: stage 1, 29 (18.5%); stage 2, 58 (36.9%); stage 3, 66 (42.0%); and stage 4, 4 (2.5%). Most of the participants, 137 (77.0%), were on ART at the time of the interview. The majority of the children, 89 (67.4%) had taken ART for less than 5 years. The ART regimens were: d4T/3TC/NVP, 50 (40.0%); AZT/3TC/NVP, 46 (36.8%); AZT/3TC/EFV, 15 (12.0%); d4T/3TC/EFV, 9 (7.2%); d4T/3TC/lopinavir/ritonavir, 4 (3.2%); and ABC/ddI/lopinavir/ritonavir, 1 (0.8%).

Caregivers were available for 160 (89.9%) of the children; the other children came by themselves. Most of the children, 99 (59.6%), had their mothers as their usual caregivers. Fathers, 20 (12.0%); grandparents, 14 (8.4%), siblings and close relatives, 24 (14.5%), and adoption centers, 9 (5.5%) were other caregivers. Most of the caregivers were HIV positive, 119 (71.7%). Of the HIV positive caregivers, 108 (93.9%) were receiving treatment. Nearly a third (32.3%) of them started HIV treatment before 15 years; the majority (89.6%) of them were on ART.

Most caregivers, 137 (77.8%) stated that disclosure of HIV status to children is important and should be done. The reasons for not wanting to disclose included: hurting child’s feelings, 25 (65.8%); may kill him/herself, 6 (15.8%); can’t bear this information, 5 (13.2%); and may refuse taking his/her drugs, 2 (5.3%). Eighty-eight (69.8%) of the caregivers preferred disclosure to children by family members other than health professionals. Most of the caregivers, 115 (89.8%), responded that disclosure should be made after 10 years of age. Eighty six (57.7%) of the caregivers reported that their children don’t ask about their illness or drugs. Telling lies, 34 (54.0%); deflecting the information, 24 (38.0%); and disclosing HIV status, 4 (6.3%) were the responses given to children who asked about their condition.

**Fig 1:**
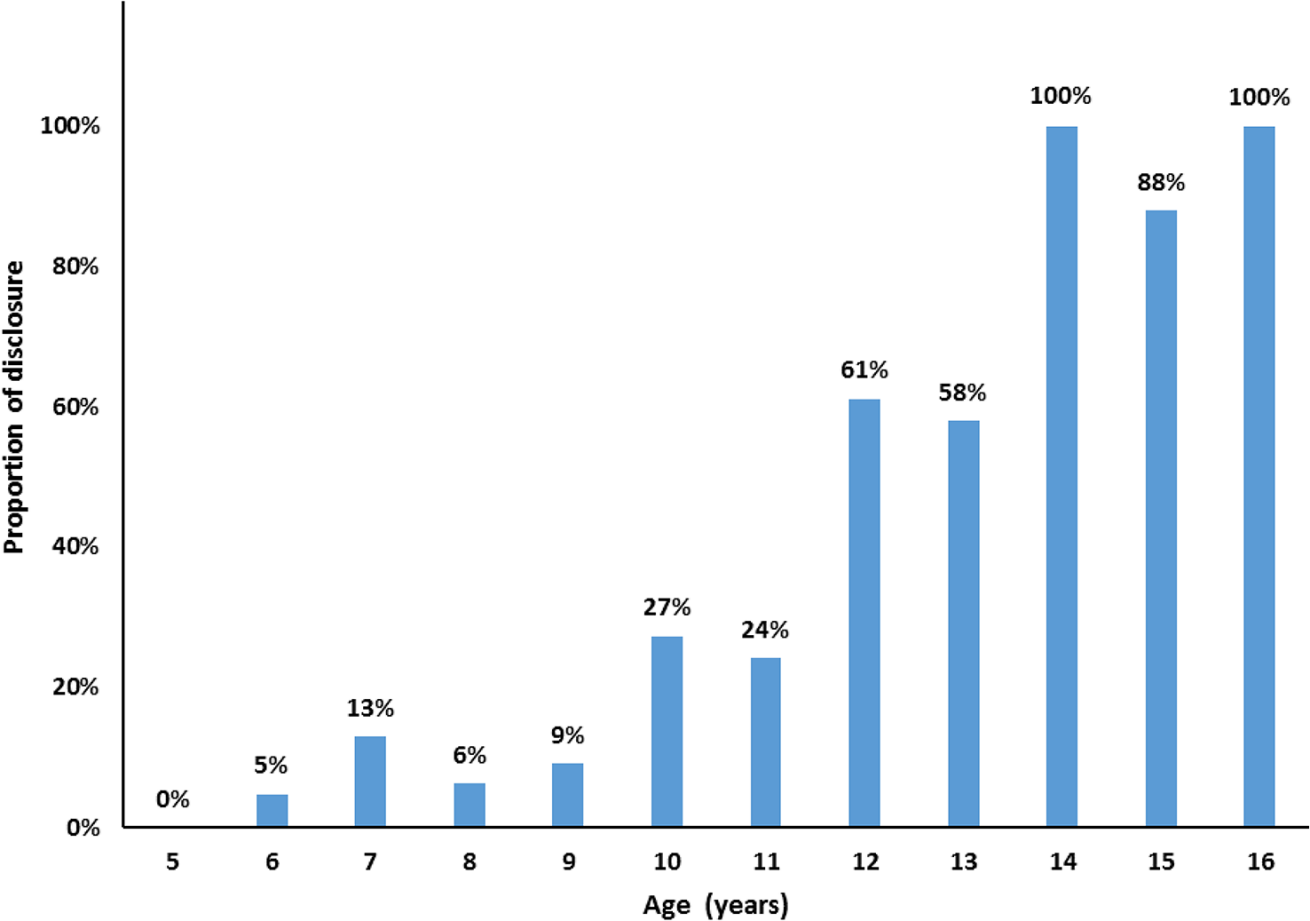
Age of participants versus the rate of disclosure at Hawassa University Referral Hospital, 2014.

The rate of HIV positive status disclosure increased as the age of children increased (Fig 1). Disclosure had been made to 59 (33.3%) of the participants. The majority (59.3%) were male. The mean age at disclosure was 9.9 years (SD = 2.4), 95%CI: 9.3–10.5. Disclosure was made by family, 38 (64.4%); health professional, 20 (33.9%); and inadvertent, 1 (1.7%). Forty-one (68.8%) of the children were already on ART during disclosure.

Caregiver and child characteristics affected the probability of disclosure of HIV status to children and adolescents. Child age more than 10 years [AOR = 6.7; 95%CI: 1.73–26.01], duration of HIV diagnosis of 5 years or more [AOR = 4.4; 95%CI: 1.26–15.06] and taking a zidovudin (AZT) based regimen [AOR = 3.5; 95%CI: 1.31–9.53] increased the odds of HIV positive status disclosure (Table 1).

**Table 1:** Association of selected child variables with disclosure of HIV status among HIV infected children, Hawassa referral hospital/Ethiopia, 2014. ART = Antiretroviral treatment; WHO = World Health Organization; OR = odds ratio.

Variable	Total	Disclosed (%)	Crude OR (95% CI)	Adjusted OR* (95% CI)
**Current age (years)**				
< 10	78	7.7	1	1
10 years or more	99	54.0	13.8 (5.50–34.75)^†^	6.7 (1.73–26.01)^¥^
**Sex of the child**				
Male	90	38.9	1.7 (0.89–3.15)	
Female	87	27.6	1	
**Grade of the child**				
Kindergarten or below	50	10.0	1	1
Elementary	127	42.5	6.7 (2.48–17.89) ^†^	1.6 (0.40–6.36)
**Birth order**				
First or second	114	33.3	1	
Third and above	55	36.4	0.9 (0.45–1.72)	
**Age HIV diagnosed (years)**				
< 5 years	90	17.8	1	1
5 years and above	68	51.5	4.9 (2.39–10.08) ^†^	2.7 (0.75–9.91)
**WHO clinical stage**				
I and II	87	27.6	1	
III and IV	70	35.7	0.7 (0.35–1.35)	
**ART started**				
Yes	137	37.2	1	
No	30	20.0	2.4 (0.91–6.19)	
**Duration on ART**				
< 5 years	89	33.7	1	
5 years and above	43	41.9	0.7 (0.33–1.49)	
**Duration since HIV diagnosis**				
< 5 years	64	21.9	1	1
5 years and above	99	40.4	2.4 (1.18–4.95) ^¥^	4.4 (1.26–15.0)^¥^
**ART regimen**				
Stavudine based	63	22.2	1	1
Zidovudine based	61	52.5	3.9 (1.77–8.41)^‡^	3.5 (1.31–9.53)^¥^

† p< 0.0001

‡ p< = 0.001

¥ p< 0.05.

* Adjusted only for significant variables in the bivariate analysis.

Length of treatment of the caregiver of more than 14 years [AOR = 3.9; 95%CI: 1.07–14.61], disclosure of caregiver’s HIV status to children and/or others [AOR = 4.7; 95%CI = 1.19–18.74], and the child’s inquiry about their condition [AOR = 4.5; 95%CI: 1.16–17.43] increased the odds of disclosure (Table 2). Educational status, employment status and HIV positive status were not associated with disclosure in the multivariable analysis. Given the associations between regimens and disclosure and age and disclosure, we evaluated the data for a potential interaction. We found that there was an association between age and zidovudine with 65.5% of children age 10 years or older on a zidovudine-based regimen versus 34.4% of those younger than 10 years old (p = 0.05). However, when we stratified by age, there was still an association with zidovudine and disclosure; in children age 10 years or older, 70% of those taking a zidovudine-based regimen were disclosed versus 41.9% disclosed in those on a stavudine regimen (p = 0.02). Similarly, in those younger than 10 years old, 19.0% of those on zidovudine were disclosed versus 3.1% of those taking a stavudine-based regimen (p = 0.07).

**Table 2:** Association of selected parent/caregiver variables with disclosure of HIV status among HIV infected children, Hawassa referral hospital/Ethiopia, 2014. ART = Antiretroviral treatment; WHO = World Health Organization; OR = odds ratio.

Variable	Total	Disclosed (%)	Crude OR (95% CI)	Adjusted OR* (95% CI)
**Age of the caregiver (years)**				
Youth (15–24)	19	21.1	1	
Adult 25–49	129	28.9	2.2 (0.52–9.27)	
Adult 50+	19	36.8	1.5 (0.48–4.90)	
**Sex of the caregiver**				
Male	30	23.3	1	
Female	136	30.1	1.4 (0.56–3.57)	
**Relationship with caregiver**				
Mother	99	32.3	1.6 (0.71–3.46)	
Father	20	25.0	1.1 (0.32–3.68)	
Other	47	23.4	1	
**Caregiver educational level**				
No education or elementary	92	29.3	1	
High school and above	70	27.1	1.1 (0.56–2.23)	
**Income of caregiver per month (USD)**				
< 30	106	30.2	1.2 (0.57–2.36)	
≥ 30	59	27.1	1	
**Occupation of caregiver**				
Employed	74	35.6	2.6 (1.21–5.59) ¥	1.8 (0.49–6.90)
Unemployed	73	17.6	1	1
**Marital status of caregiver**				
Married	99	27.3	1	
Unmarried	62	32.3	1.3 (0.64–2.54)	
**Caregiver HIV status**				
Positive	119	29.4	1.1 (0.51–2.31)	
Negative or Unknown	47	27.7	1	
**Caregiver Care and Treatment**				
Yes	108	29.6	1.1 (0.19–5.71)	
No	7	28.6	1	
**Timing of Care and Treatment**				
Before 2000	32	43.8	3.2 (1.28–8.14) ¥	3.9 (1.07–14.61) ¥
2000 and after	67	19.4	1	1
**Caregiver is on ART**				
Yes	103	32.0	5.2 (0.64–41.87)	
No	12	8.3	1	
**Duration of ART**				
Before 2000	27	37.0	1.9 (0.73–5.08)	
2000 and after	64	23.4	1	
**Caregiver disclosed status**				
Yes	99	34.3	NA	
No	14	0		
**To whom did you disclose**				
Others only	49	22.4	1	1
Child only +/- others	49	46.9	3.1 (1.27–7.33) ¥	4.7 (1.19–18.74) ¥
**Does the child ask about HIV**				
Yes	63	11.1	1	1
No	86	27.9	3.1 (1.24–7.74) ¥	4.5 (1.16–17.43)¥

† p < 0.0001

‡ p< = 0.001

¥ p< 0.05.

* Adjusted only for significant variables in the bivariate analysis.

Of caregivers who did not disclose, a small majority thought that disclosure should be done by families and not healthcare providers (Table 3). Most of these caregivers, 79 (66.9%), believed that children would not understand the discussion on HIV, and that not discussing HIV keeps death away, 69 (60.5%). Also, a majority of caregivers strongly disagreed with the notion that disclosure might improve school performance, 63 (53.8%) (Table 3).

**Table 3:** Beliefs of caregivers who have not disclosed the HIV positive status to children and adolescents regarding HIV positive disclosure (N = 118).

Questions	Strongly Agree (%)	Agree (%)	Disagree (%)	Strongly Disagree (%)
Child doesn’t understand discussion about HIV?	79(66.9)	3(2.5)	3(2.5)	33(28.0)
Avoiding thinking about HIV keeps death away?	69(60.5)	20(17.5)	2(1.8)	23(20.2)
Children don’t keep secrets?	55(46.6)	8(6.4)	2(1.7)	52(44.1)
Disclosure of HIV positive status to children improves their adherence?	58(50.0)	12(10.3)	5(4.3)	41(35.3)
Disclosure of HIV positive status to children improves their school performance?	35(29.9)	10(8.5)	8(6.8)	63(53.8)
Inadvertent disclosure hurts the feeling of children?	97(83.6)	9(7.8)	2(1.7)	8(6.9)
Disclosure should be done by doctors or nurses?	48(41.4)	16(13.8)	3(2.6)	49(42.2)
Disclosure should be done by families?	66(58.4)	17(15.0)	-	29(25.7)

## Discussion

With the introduction of pediatric ART, the lifespan of the affected children has increased bringing issues of HIV positive status disclosure to the agenda. Disclosure of HIV positive status to children is generally considered as a complicated issue with emotional involvement of both the caregivers and health professionals [5]. However, few studies have tried to establish the positive impacts of HIV positive status disclosure on the health outcomes of the children [8, 9].

In the current study, disclosure was made to only a third of the participants. In a systematic review by Vreeman *et al*, the rate of disclosure widely ranged from 0% to 69.2% [10]. This finding is an indication that the rate of disclosure depends largely on local contexts which should be explored. The mean age at disclosure was nearly 10 years which is younger than in the study by Butler *et al* [5].

Most of the predictors of disclosure identified in the current study were also reported elsewhere. Older child age [11, 12], longer duration on ART [13, 14], disclosure of care giver HIV status to their children and/or others [15–17], and if children were asking about their health condition [18, 19] were independently associated with an increased odds of disclosure. We also found that taking zidovudine-containing ART regimens was independently associated with disclosure. While age was associated with zidovudine-containing regimens, we still found an association after stratifying by age suggesting that the association is not explained by an interaction with age. This might be explained by the differences in tolerability and side effects of different regimens; the hypothesis however would have to be that parents of children on stavudine think it is causing such side effects that the parents do not want to disclose for fear the child will resist taking the medicine. There are no data to support this hypothesis currently though this warrants further investigation. Finally, another possible explanation is an unmeasured confounder of provider. It is possible that a provider who prescribes zidovudine more often at the clinic also somehow facilitates more disclosure than providers prescribing stavudine.

Being on ART or having symptomatic disease with an advanced WHO clinical stage might be expected to facilitate HIV positive status disclosure to children and adolescents. The assumption would be that taking drugs for a long time or having recurrent or persistent symptoms of disease would bring up questions regarding their health in the child and adolescent. However, we found that in this setting, neither of these factors was found to facilitate HIV positive status disclosure. This underlines the fact that sociocultural determinants are more important if addressed properly. The finding that most caregivers think that children don’t keep secrets, and that disclosure may hurt their feelings reveal some of the social and emotional challenges in addressing disclosure.

Although the majority of the caregivers believed that disclosure should be made, we found that the majority of the children were not disclosed. This finding is in agreement with the findings of Heeren *et al* in South Africa [1]. The findings clearly show that only caregiver knowledge is not adequate to facilitate disclosure of HIV positive status to children and adolescents. Further exploration of effective strategies for increasing the rate of disclosure is warranted through identification of factors that might hinder it in the specific local context.

The reasons for non-disclosure were given by a few of the participants. Most of the mentioned reasons are related to fear of the negative effects of disclosure by the caregivers. Similar finding was reported in a systematic review by Vreeman *et al* [7]. The negative effects of disclosure were considered by the caregivers to be related to the child’s health outcomes or the stigma to themselves thinking that the child might not keep secrets which is also the case in the current study.

The limitation of our study is that it employed a quantitative approach to collect data, and the reasons for non-disclosure from the caregivers’ perspective couldn’t be explored in depth. A qualitative approach with in-depth interview and focus group discussions are recommended. For effective educational interventions, knowing the predictors and the reasons given by the caregivers is imperative.

In conclusion, our findings showed that the rate of disclosure among HIV infected children and adolescents in this setting is low. Various child and caregiver factors were associated with the probability of HIV positive status disclosure to children and adolescents. We recommend educational strategies for caregivers, adolescents and healthcare professionals to address these determinants. Synchronously, qualitative studies that specifically explore the perceived barriers and facilitators of disclosure should be done to provide better insight into this complex process. The association of the zidovudine with disclosure should be evaluated in a separate population.

## Supporting Information

S1 Questionnaire The questionnaire used in the collection of data as described. (DOCX)

S1 Dataset The full de-identified dataset with variable labels. (CSV)
