# Supplementary material for: Cross Sectional Characterization of Factors Associated with Pediatric HIV Status Disclosure in Southern Ethiopia
Source: PLoS One. 2015 Jul 13;10(7):e0132691. doi: 10.1371/journal.pone.0132691 (PMC4500496; doi:10.1371/journal.pone.0132691)
Supplement: S1 Questionnaire — (DOCX) [file pone.0132691.s001.docx]

| **Code** | Who is doing the interview? | 1. Patient 2. Caregiver | | | | |  | | | |
| --- | --- | --- | --- | --- | --- | --- | --- | --- | --- | --- |
| 1. **Child Socio-demographics** | | | | | | | | | | |
| 101 | Age | _________________ | | | | |  | | | |
| 102 | Sex | 1. Male  2. Female | | | | |  | | | |
| 103 | Grade |  | | | | |  | | | |
| 104 | Birth Order | _________________ | | | | |  | | | |
| 105 | Address |  | | | | |  | | | |
| **2. Caregiver Socio-demographics** | | | | | | | | | | |
| 201 | Age | ------------------------ | | | | |  | | | |
| 202 | Sex | 1. Male  2. Female | | | | |  | | | |
| 203 | Relationship with Child | _____________________ | | | | |  | | | |
| 204 | Education | _____________________ | | | | |  | | | |
| 205 | Income |  | | | | |  | | | |
| 206 | Occupation | -------------------------------------- | | | | |  | | | |
| 207 | Marital Status | 1. Married  2. Widow  3. Other____________________ | | | | |  | | | |
| **3. Child HIV and ART Information** | | | | | | | | | | |
| 301 | Diagnosis when? | ------------ | | | | |  | | | |
| 302 | WHO clinical HIV stage | ______________________ | | | | |  | | | |
| 303 | Prophylaxis? | 1. Yes  2. No | | | | |  | | | |
| 304 | ART started? | 1. Yes  2. No | | | | |  | | | |
| 305 | If Yes, when? | ___________________ | | | | |  | | | |
| 306 | Regimen? | _________________________ | | | | |  | | | |
| 308 | Early infant feeding | 1. Exclusive breast feeding  2. Exclusive formula feeding  3. Mixed  4. Other___________________ | | | | |  | | | |
| 309 | Total duration of breast feeding | __________________ | | | | |  | | | |
| **4. Caregiver HIV/ART Status** | | | | | | | | | | |
| 401 | HIV status | ________________________ | | | | |  | | | |
| 402 | If positive : |  | | | | |  | | | |
|  | When did you know your status? | ________________________ | | | | |  | | | |
|  | HIV Care | 1. Yes  2. No | | | | |  | | | |
|  | If yes, since when? |  | | | | |  | | | |
|  | ART? | 1. Yes  2. No | | | | |  | | | |
|  | If yes, since when? |  | | | | |  | | | |
| 403 | Did you disclose your status to any other person? | 1. Yes  2. No | | | | |  | | | |
| 404 | If yes, to whom? | 1. Spouse  2. Children  3. Relative  4. Other___________ | | | | |  | | | |
| 405 | If not disclosed, why? | ________________________________ | | | | |  | | | |
| **5. Child HIV status Disclosure** | | | | | | | | | | |
| 501 | Do you think it is important to disclose to the child? | 1. Yes  2. No | | | | | | |  | |
| 502 | If No: |  | | | | | | |  | |
|  | Why? | __________________________ | | | | | | |  | |
| 503 | If Yes: |  | | | | | | |  | |
|  | Why? |  | | | | | | |  | |
|  | Who should do it? |  | | | | | | |  | |
|  | At what age? |  | | | | | | |  | |
| 504 | Does your child ask you about his drugs/condition? | 1. Yes  2. No | | | | | | |  | |
| 505 | What do you tell him? | 1. I deflect the information 2. I tell him a lie 3. I tell him that he has HIV 4. Other_______________________________________________________ | | | | | | |  | |
| 506 | Does s/he resist taking drugs? | 1. Yes  2. No | | | | | | |  | |
| 507 | Did you disclose your child’s HIV status to her/himself? | 1. Yes 2. No | | | | | | |  | |
| 508 | If yes, when? | _________________ | | | | | | |  | |
| 509 | At what age? | __________________ | | | | | | |  | |
| 510 | Who made the disclosure? | 1. Family 2. Health professionals 3. Inadvertent 4. Other_____________ | | | | | | |  | |
| 511 | Was s/he on ART during disclosure? | 1. Yes 2. No | | | | | | |  | |
| 512 | If Yes to Q 511, how long before the disclosure and after the disclosure? | _______________________________  _______________________________  _______________________________ | | | | | | |  | |
| 513 | When do you think is the appropriate age to disclose HIV status to the child? |  | | | | | | |  | |
|  | If no to Q 507, why? GO TO QUESTION NUMBER 514. | | | | | | | | | |
| If the answer to 507 is NO, could answer the following possible reasons for not disclosing: | | | | | | | | | | |
|  | | 1=Strongly Disagree | | 2=Disagree | | 3=Agree | | | 4=Strongly Agree | |
| 514 | Child doesn’t understand discussion about HIV |  | |  | |  | | |  | |
| 515 | I didn’t disclose because of my illness |  | |  | |  | | |  | |
| 516 | Avoiding thinking about HIV keeps death away. |  | |  | |  | | |  | |
| 517 | Children don’t keep secrets and tell secrets to others |  | |  | |  | | |  | |
| 518 | Disclosure may improve their adherence. |  | |  | |  | | |  | |
| 519 | Disclosure may improve their school performance. |  | |  | |  | | |  | |
| 520 | Inadvertent disclosure may hurt the child’s feeling |  | |  | |  | | |  | |
| 521 | Disclosure should be done by doctors/nurses |  | |  | |  | | |  | |
| 522 | Disclosure should be by family |  | |  | |  | | |  | |
| **6.ONLY FOR CHILDREN WHO WERE DISCLOSED:** | | | | | | | | | | |
| 601 | Did you witness any changes after his/her HIV status was disclosed to him/her? | | | | | | | | | |
|  |  | 1=Worsened | | 2=No change | | 3=Improved | | | 4= Improved much | |
|  | School Performance |  | |  | |  | | |  | |
|  | School Attendance |  | |  | |  | | |  | |
|  | Taking His pills |  | |  | |  | | |  | |
|  | Questions Regarding his health |  | |  | |  | | |  | |
|  | Behavior/cooperation |  | |  | |  | | |  | |
|  | Depression |  | |  | |  | | |  | |
|  |  |  | |  | |  | | |  | |
| 602 | Before and After Disclosure | ***Consecutive*** three measurements **Before** | | | | ***Consecutive*** three measurements **After** | | | | |
|  |  | 1 | 2 | | 3 | 1 | | 2 | | 3 |
|  | CD4 Count |  |  | |  |  | |  | |  |
|  | Weight (KG) |  |  | |  |  | |  | |  |
|  | Height (cm) |  |  | |  |  | |  | |  |
| 603 | Self- Reported Adherence |  | | | | | | | | |
